# Comparison of clinical outcomes following osteochondral allograft transplantation for osteochondral versus chondral defects in the knee

**DOI:** 10.1186/s43019-022-00149-z

**Published:** 2022-05-04

**Authors:** John Reza Matthews, Joseph Brutico, Jeremy Heard, Kashyap Chauhan, Bradford Tucker, Kevin Blake Freedman

**Affiliations:** 1 Department of Orthopedic Surgery, Thomas Jefferson Rothman Institute Sports Fellow, 925 Chestnut St., Philadelphia, PA 19107 USA; 2 Department of Orthopedic Surgery, Rothman Institute Research Fellow, Philadelphia, USA; 3 Internal Medicine Resident, Thomas Jefferson University, Philadelphia, USA; 4 Cartilage Restoration Center, Orthopaedic Surgery, Thomas Jefferson Rothman Institute, Philadelphia, USA

**Keywords:** Osteochondral allograft transplantation, Articular cartilage defect, Cartilage restoration, Allograft

## Abstract

**Purpose:**

Osteochondral allograft (OCA) transplantation is a restorative technique for addressing articular cartilage defects by transferring mature viable chondrocytes with subchondral bone into size-matched lesions. The purpose of this study was to compare differences in clinical and functional outcomes in patients treated with OCA for osteochondral defects compared with isolated chondral pathology.

**Methods:**

A retrospective review identified patients who underwent OCA transplantation and grouped them into osteochondral or isolated chondral pathology. Demographic data, surgical history, lesion characteristics, complications, and rate of subsequent surgery were reviewed. The review included 86 patients (24 osteochondral, 62 chondral) with a mean follow-up of 5.4 ± 1.4 years. Outcome measures included the Knee Injury and Osteoarthritis Outcome Score for Joint Replacement (KOOS, JR.), International Knee Documentation Committee (IKDC), and Short Form Health Survey (SF-12) physical scores. Failure was defined to include revision OCA, graft removal, conversion to ACI, or conversion to arthroplasty.

**Results:**

The average age at surgery was 32.3 and 37.3 years for the osteochondral and chondral groups, respectively (*P* = 0.056). The medial femoral condyle was the most common defect location in both groups. *P* < 0.05 was considered statistically significant. Patients with osteochondral pathology had significantly greater KOOS JR., IKDC, and SF-12 scores (*P* < 0.05), and fewer failures were reported in the osteochondral group (8.3% versus 32.3%, *P* = 0.045). When controlling for age, sex, laterality, BMI, and presence of a concomitant procedure, patients with osteochondral pathology were found to have better KOOS and IKDC scores, but there was no difference in SF12 scores or rates of failure between groups.

**Conclusion:**

The findings of this study indicate that patients undergoing OCA for osteochondral defects may have greater functional outcomes and similar failure rates compared with OCA transplantation for isolated chondral pathology.

## Introduction

Chondral lesions are commonly encountered during knee arthroscopic surgery, with a reported prevalence of 63–66% [1]. Articular cartilage lesions can cause pain, recurrent effusions, and functional impairment [2, 3]. Previous studies have demonstrated that unaddressed lesions or excised fragments result in poor knee function and osteoarthritis [4]. There are several options available to address focal chondral and osteochondral defects, including palliative options, such as chondroplasty, reparative with microfracture or fragment fixation, and restorative options, such as autologous chondrocyte implantation (ACI), osteochondral autograft transfer (OAT), or osteochondral allograft (OCA) transplantation [5–7]. Ultimately, treatment selection depends on a variety of factors including lesion characteristics, patient age, function, and concomitant pathology. Previous studies have demonstrated that microfracture has the best results in lesions < 2 cm^2^, whereas lesions between 2 and 4 cm^2^ can be addressed with ACI, OATs, or allograft transplantation, and lesions > 4 cm^2^ with ACI or OCA [2, 13–15] Generally speaking, in lesions > 2 cm^2^ where bone loss is present, osteochondral allograft can be a first-line treatment [16].

The use of OCA has increased in recent years as new studies have demonstrated satisfactory long-term outcomes with 5- and 10-year survival rates of 95% and 85%, respectively [8–11]. However, there are several limitations to the use of OCA, including donor tissue availability, contour matching, limited time from graft harvest to implantation, and potential for disease transmission [8, 12]. In large uncontained chondral defects, defects with sclerotic subchondral bone, or subchondral bone loss, osteochondral allograft may be a clearer choice. However, the use of OCA in focal articular cartilage loss without subchondral bone involvement is more controversial. The purpose of this study was to compare differences between OCA for osteochondral defects compared with focal chondral lesions to determine differences in outcomes and complications. We hypothesized OCA for isolated chondral compared with osteochondral defects would result in similar outcomes.

## Materials and methods

After institutional review board approval (#0153), patients who underwent OCA from January 2012 to December 2016 at a single institution were identified via a database query. A retrospective chart review of each patient’s clinical and surgical history was performed, and patients with more than 2 years follow-up were included. Patients were excluded if their medical history revealed absence of 50% or more of the ipsilateral meniscus, inflammatory arthropathy, or incomplete preoperative or intraoperative history. A total of 118 patients were eligible for the study, but 32 were lost to follow-up, leaving 86 patients for final analysis. Patients were grouped according to disease etiopathogenesis. Those with a diagnosis of osteochondritis dissecans or avascular necrosis were placed into the osteochondral group, while the chondral group had a diagnosis of trauma or focal degenerative defect.

Twenty-four (27.9%) patients were diagnosed with osteochondral pathology, 22 (91.7%) from osteochondritis dissecans and 2 (8.3%) secondary to avascular necrosis. Sixty-two (72.1%) patients had isolated chondral pathology, further subdivided into acute trauma (33 patients, 53.2%), focal degenerative defect (27 patients, 43.6%), and chondromalacia of the trochlea (1 patient, 1.6%) and patella (1 patient, 1.6%). Demographic data, including age, laterality, sex, and body mass index (BMI), were recorded (Table 1).

**Table 1:** Comparison of demographic data between osteochondral and chondral lesions

	Osteochondral	Chondral	*P*-value
	*N* = 24	*N* = 62	
Age at surgery, years	32.25 ± 11.06	37.30 ± 10.77	0.056
Sex			
Male	17 (70.83%)	31 (50.00%)	0.081
Female	7 (29.17%)	31 (50.00%)	
BMI, kg/m^2^	26.43	29.17	0.006*
Laterality			
Right	15 (62.50%)	26 (41.94%)	0.098
Left	9 (37.50%)	36 (58.06%)	
Etiology			
AVN	2 (8.3%)		
OCD	22 (91.6%)		
Acute trauma		33 (53.2%)	
Focal DJD		27 (43.6%)	
Chondromalacia patella		1 (1.6%)	
Chondromalacia trochlea		1 (1.6%)	
Grade			
2	–	1 (1.66%)	
3	2 (9.52%)	10 (16.66%)	
4	19 (90.48%)	49 (81.66%)	
Mean	3.90	3.80	0.234

*BMI* body mass index, *AVN* avascular necrosis, *OCD* osteochondritis dissecans, *DJD* degenerative joint disease. *Values significant at *P* < 0.05

In our practice, most patients with osteochondral pathology from osteochondritis dissecans are treated with fresh OCA. For those with chondral pathology only, it was at the surgeon’s discretion to perform fresh OCA as opposed to an alternative treatment option such as autologous chondrocyte implantation for the defect. Overall, OCA transplantation was indicated for symptomatic younger patients with magnetic resonance imaging (MRI) evidence of focal defects measuring > 2 cm^2^ that failed to improve with conservative management or patients requiring revision procedures if there was no radiographic evidence of Kellgren–Lawrence grade 3 or 4 osteoarthritis. Full-length standing lower extremity x-rays were obtained only in patients with clinical evidence of varus/valgus malalignment requiring osteotomy. There was one patient in our study who required a high tibial osteotomy with concomitant cartilage restoration. The OCA transplantation surgery was performed through a medial or lateral parapatellar arthrotomy using a press-fit cylindrical graft matched to the defect size. All preoperative MRIs were reviewed for degree of pathology, lesion size, location, and involvement of subchondral bone. The operative notes were also reviewed for similar details in addition any concomitant procedures performed (Table 2). In total, 9 patients in the osteochondral group and 32 in the chondral group had concomitant procedures. The mean lesion grade in the osteochondral group was 3.9 compared with 3.8 for the chondral group (*P* = 0.234). Pathology was grouped into four groups: condylar (medial or lateral), patellar, trochlear (medial or lateral), and multifocal.

**Table 2:** Comparison of concomitant procedures between osteochondral and chondral lesions

	Osteochondral (*N* = 9)	Chondral (*N* = 32)
ACI	1 (11.1%)	–
ACLR	–	3 (9.4%)
ACLR + meniscal repair	–	1 (3.1%)
ACLR + meniscal transplant	–	2 (6.2%)
ACLR + TTO + meniscal repair	–	1 (3.1%)
Curettage and bone grafting	1 (11.1%)	–
Debridement/synovectomy	1 (11.1%)	3 (9.4%)
Fulkerson TTO	1 (11.1%)	7 (21.9%)
Fulkerson TTO + MPFLR	–	3 (9.4%)
High tibial osteotomy	1 (11.1%)	–
Lateral retinacular lengthening	–	1 (3.1%)
Meniscal transplant	1 (11.1%)	1 (3.1%)
Meniscectomy	1 (11.1%)	2 (6.3%)
Microfracture	2 (22.2%)	–
MPFLR + lateral retinacular lengthening	–	5 (15.6%)
ROH	–	2 (6.3%)
TTO + meniscectomy	–	1

*ACI* autologous chondrocyte implantation, *ACLR* anterior cruciate reconstruction, *TTO* tibial tubercle osteotomy, *MPFLR* medial patellofemoral ligament reconstruction, *ROH* removal of hardware. Debridement includes lysis of adhesions and chondroplasty. Results reported as *N* (%)

Outcome measures included the Knee Injury and Osteoarthritis Outcome Scores for Joint Replacement (KOOS JR.), International Knee Documentation Committee (IKDC) evaluation, and Short Form Health Survey (SF-12) physical scores. In addition, the need for subsequent surgery, complications, and failure rates were recorded. Failure was defined to include revision OCA, graft removal, conversion to ACI, or conversion to arthroplasty.

Summary statistics, including mean and standard deviation, were calculated. The Shapiro–Wilk test was used to determine normality of the data. The Mann–Whitney *U* test was used to compare the means of two unpaired groups for nonparametric data. The Kruskal–Wallis test was used when comparing continuous data for two groups with nonparametric data. The Fischer exact test was used for categorical data. Statistical significance was set to *P* < 0.05. A regression analysis was performed comparing failures and outcome measures while adjusting for the confounding variables of, age, sex, laterality, BMI, and presence of a concomitant procedure. Further, to control for location of lesion, a separate univariate analysis was performed on the group of patients with lesions localized to the medial or femoral condyle.

## Results

### Patient demographics

**Fig. 1:**
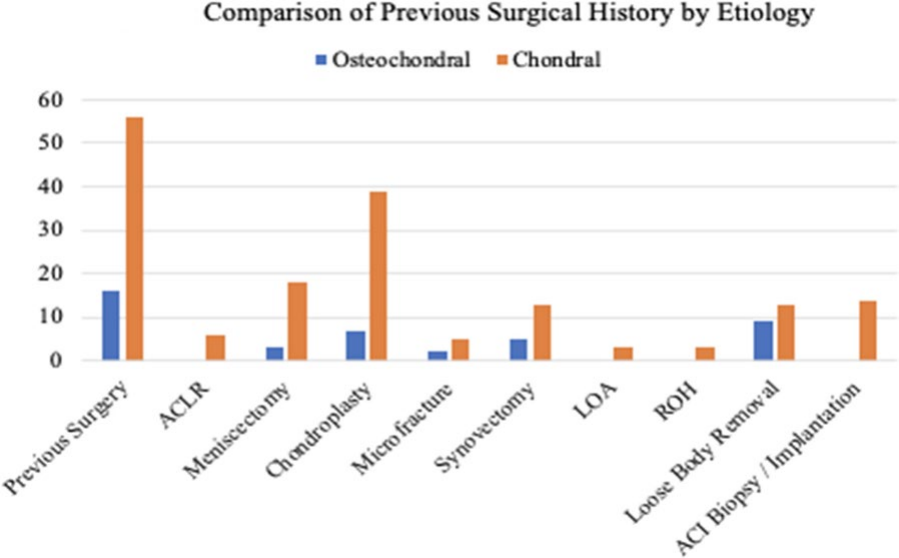
Comparison of previous surgeries by etiology. *ACLR* anterior cruciate ligament reconstruction, *LOA* lysis of adhesions, *ROH* removal of hardware, *ACI* autologous chondrocyte implantation

The patient population was composed of 48 (55.8%) males and 38 (44.2%) females, with an average follow-up of 5.4 ± 1.4 years. In the osteochondral group 70.8% were male, compared with 50% in the chondral group (*P* = 0.133). The mean age at surgery in patients with osteochondral lesions was 32.3 ± 11.1 years, compared with 37.3 ± 10.8 years in patients of chondral etiology (*P* = 0.067). The mean BMI (kg/m^2^) was 26.4 in the osteochondral group and 29.2 in the chondral group (*P* = 0.068). Nine (37.5%) patients in the osteochondral group underwent a concomitant procedure, compared with 32 (51.6%) in the chondral group (*P* = 0.350). A total of 56 patients (90.3%) in the chondral group and 16 patients (66.6%) in the osteochondral group underwent a surgical procedure on their affected knee prior to OCA transplantation (Fig. 1). The rate of previous surgery was significantly greater in the chondral group (*P* = 0.018).

The defect location and area is presented Table 3. The difference in laterality between groups was not statistically significant (*P* = 0.141). In both groups, the medial femoral condyle was the most frequent site of injury (74.2% osteochondral group versus 45.0% chondral group), and the lateral trochlea was the least frequent (3.2% osteochondral versus 7.3% chondral group). A total of 7 (29.2%) patients in the osteochondral group and 27 (43.5%) patients in the chondral group were found to have lesions in multiple locations (*P* = 0.221). After grouping lesions of the medial and lateral condyles for the purpose of univariate subgroup analysis, there were 19 patients in the osteochondral group (39.6%) and 29 patients in the chondral group (60.4%).

**Table 3:** Comparison of lesion location and size (mm^2^) between osteochondral and chondral defects

	Osteochondral	Chondral	*P*-value
Medial femoral condyle	23 (74.2%), 501.28 ± 343.33 mm^2^	49 (45.0%), 487.55 ± 240.71 mm^2^	0.865
Lateral femoral condyle	4 (12.9%), 476.56 ± 96.34 mm^2^	17 (15.6%), 443.13 ± 286.03 mm^2^	0.825
Patella	2 (6.5%), 412.00 ± 124.45 mm^2^	24 (22.0%), 391.50 ± 193.34 mm^2^	0.886
Medial trochlea	1 (3.2%), 625.00 ± 0.00 mm^2^	11 (10.1%), 259.70 ± 150.28 mm^2^	–
Lateral trochlea	1 (3.2%), 450.00 ± 0.00 mm^2^	8 (7.3%), 308.25 ± 111.34 mm^2^	–

Results reported as mean ± standard deviation. ^−^Indicates group sizes are too small for adequate comparison between groups

### Patient reported outcomes

**Fig. 2:**
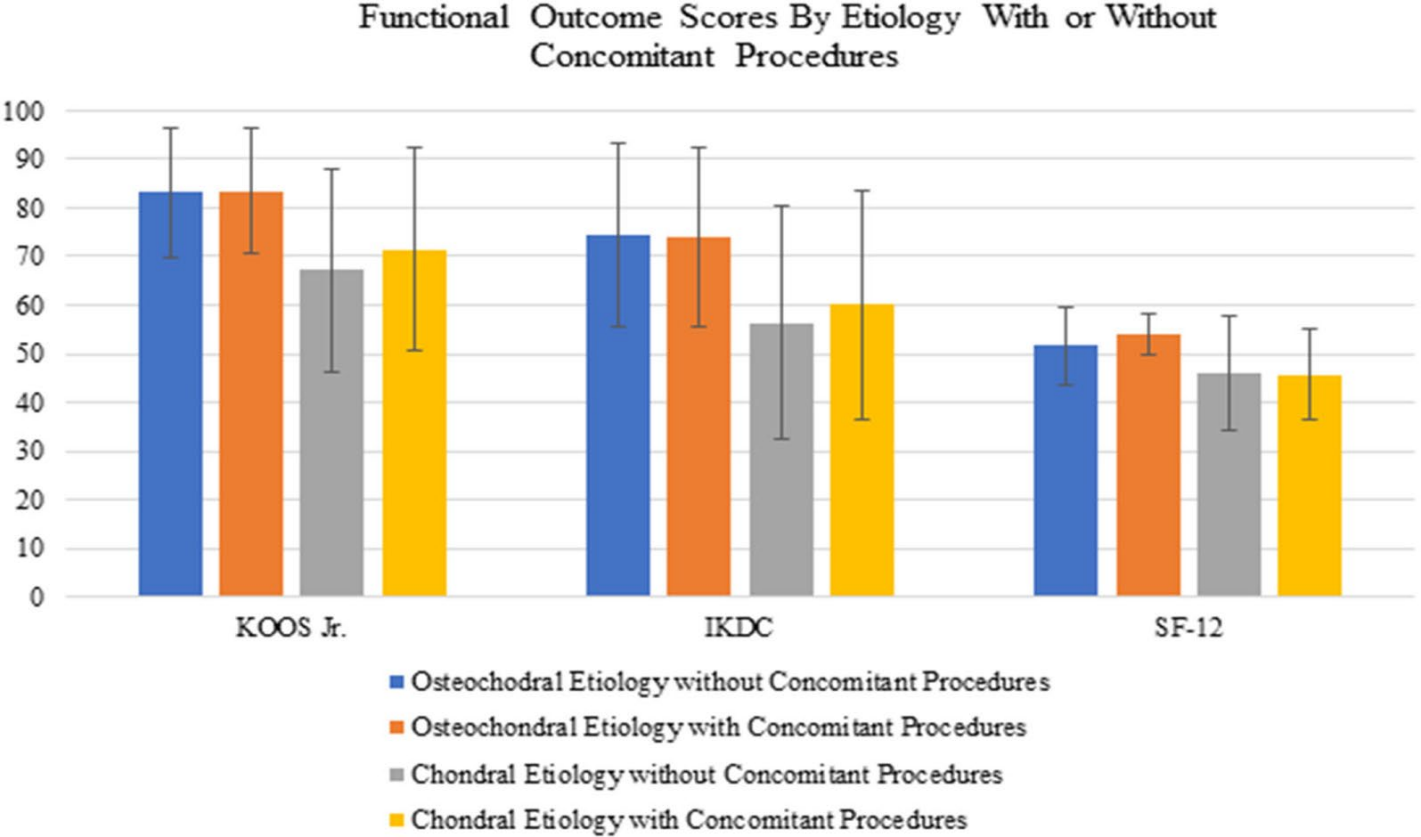
Comparison of functional outcome by etiology in which OCA was performed with or without concomitant procedures. KOOS, JR., Knee injury and Osteoarthritis Outcome Score for Joint Replacement; IKDC, International Knee Documentation Committee; SF-12P, Short Form Health Survey Physical

At final follow-up, patients who underwent OCA transplantation for osteochondral pathology reported significantly greater functional outcome scores compared with those with chondral pathology. The mean KOOS JR. score was 83.2 for the osteochondral group and 69.4 for the chondral group (*P* = 0.005). The mean IKDC scores were 74.2 and 58.3 for the osteochondral and chondral groups (*P* = 0.007), respectively. The osteochondral group had a mean SF-12 score of 50.4 compared with a mean of 44.4 (*P* = 0.003) for the chondral group. Among patients with lesions localized to the medial or lateral condyle only, the osteochondral group reported superior scores on the KOOS JR. (*P* = 0.047), IKDC (*P* = 0.037), and SF-12 (*P* = 0.011). Further, comparison between the chondral group with concomitant procedures and those without did not demonstrate a difference, as seen in Fig. 2 (KOOS JR. *P* = 0.25, IKDC *P* = 0.31, SF-12 *P* = 0.17).

Regression analysis controlling for age, sex, BMI, laterality, and presence of a concomitant procedure found that patients with osteochondral pathology had better KOOS JR. (*P* = 0.003) and IKDC (*P* = 0.008) scores, but there was no significant difference in SF-12 scores. Among the subgroup of patients with condylar lesions only, patients with osteochondral pathology had better KOOS JR. scores (*P* = 0.034), but there was no significant difference in IKDC or SF-12 scores.

### Failures and revisions

There were 20 (32.3%) failures in the chondral group compared with 2 (8.3%) in the osteochondral group (*P* = 0.045). Among the subgroup of patients with condylar lesions only, there was one failure in the osteochondral group and nine failures in the chondral group (*P* = 0.065). Regression analysis found that that the difference in failure rates between groups was not statistically significant (*P* = 0.164).

Of the two patients who failed treatment in the osteochondral group, both underwent revision OCAs and one eventually progressed to total knee arthroplasty (TKA). Of the 20 failures in the chondral group, 4 progressed to autologous chondrocyte implantation and 9 progressed to total (7) or partial (2) knee arthroplasty. There were four patients who underwent revision OCAs, and one who underwent microfracture due to avascular necrosis of the allograft plug. One patient, who initially received a five-plug allograft transplantation, required subsequent loose body removal and chondroplasty because one of the five plugs had failed and was floating in the joint space. Finally, there was one patient who developed a subchondral cyst that required lysis of adhesions and retrograde drilling of the patella.

## Discussion

This study evaluated the clinical and functional results of patients following osteochondral allograft transplantation for osteochondral defects compared with isolated chondral pathology. Patients with osteochondral defects treated with OCA reported superior KOOS JR., IKDC, and SF-12 scores compared with the isolated chondral group. However, this effect was only seen in the KOOS JR. and IKDC scores after regression.

Regarding the subgroup of patients with condylar lesions, regression analysis demonstrated that patients with osteochondral pathology had better KOOS JR. scores, but there was no significant difference in IKDC or SF-12 scores. These results suggest that patients who undergo OCA for the treatment of osteochondral defects may perform better than patients treated for isolated chondral pathology, but further research is needed to clarify this difference. Previous studies have supported the efficacy of OCA for OCD [17, 18], AVN [19] and a variety of pathologies involving articular cartilage [18, 20], but this is the first study comparing OCA for the treatment of osteochondral and chondral lesions.

Demographic data, including age, sex, BMI, laterality, and the presence of a concomitant procedure, were not significantly different between groups. Differences in lesion location and grade were also reviewed to identify confounding factors contributing to the superior outcomes seen in the osteochondral group. Previous studies have demonstrated that lesion location is strongly associated with postoperative outcomes [21, 22]. The chondral cohort in our study had a higher percentage of patellofemoral pathology (39.4%) compared with the osteochondral group (12.9%). Gracitelli et al [23] demonstrated worse survivorship and functional outcomes of patellar OCAs at 5 and 10 years when compared with outcomes of femoral condyle OCAs reported by Emmerson et al. [18]. However, the latter study did not differentiate between OCA treatment for chondral or osteochondral etiology [18]. Therefore, the higher propensity of patellofemoral defects in the chondral group may be a confounder to the inferior clinical outcomes seen. In contrast, it is unlikely that lesion grade was the driving factor for the superior outcomes reported in the osteochondral group. While the osteochondral group had a higher lesion grade (3.9 versus 3.8), the difference was not statistically significant (*P* = 0.234), and previous studies have demonstrated that lesion grade does not impact clinical outcomes [24].

The number of operations prior to OCA may have contributed to the difference in functional outcome scores. Frank et al. found previous surgical procedures to be an independent predictor of OCA failure [9]. In the present study, 56 (90.3%) patients in the chondral cohort underwent prior surgery compared with 16 (66.6%) in the osteochondral group (*P* = 0.018), with the majority related to chondroplasty and partial meniscectomy. Although there were no statistically significant differences with regard to the proportion of patients who underwent a concomitant procedure between the two groups, 12 (37.5%) patients in the chondral group required a tibial tubercle osteotomy compared with only one (11.1%) patient in the osteochondral group. There were also a greater number of ligamentous reconstructions involving the ACL and MPFL in the chondral group (15 patients) compared with the osteochondral group (0 patients), which contributed to the lower functional outcome scores seen in the chondral cohort.

This study has several limitations. First, there were several eligible patients who were lost to follow-up. In addition, some patients may have sought surgical care at another institution without our knowledge, resulting in an underestimation of reoperation and/or failure rates. The number of patients lost to follow-up was similar for both groups. There is also the potential for transfer bias as patients with worse outcomes are more likely to return for care. The lack of long-term imaging precluded additional investigation into graft healing and integrity as well as progression and evaluation of arthritis. The retrospective study design did not allow for group randomization and similar patient characteristics. Although similar, the demographic data between the two populations were not identical. There were also a higher number of patients in the chondral group compared with the osteochondral group with variable etiopathogenesis, and defect location. Additionally, long-standing x-rays were not routinely ordered, and differences in hip–knee–ankle angles were unable to be assessed. There was also an insufficient number of preoperative outcome scores in both groups. However, lesion size, laterality, and grade were similar. Additionally, analysis of postoperative radiographic healing was not possible.

There were a large number of patients with concomitant pathology addressed at the time of OCA transplantation, making it difficult to perform direct comparisons between cohorts. An attempt was made to perform a subgroup analysis of the concomitant procedures, but the sample sizes were too small for meaningful comparison. Our study is at risk for type II error due to small sample size. Last, this study included only patients treated by surgeons at one institution that performs a high volume of OCAs, which may introduce performance bias.

## Conclusion

The findings of this study indicate that patients undergoing OCA for osteochondral defects may have greater functional outcomes with similar failure rates compared with OCA transplantation for isolated chondral pathology.

## Data Availability

All raw data are stored and available for review.
